# List of JECT reviewers 2024

**DOI:** 10.1051/ject/2024035

**Published:** 2024-12-20

The Editorial Team would like to thank this diverse and international team of peer reviewers for reviewing at least one article for JECT over the past year. Your contributions have substantially improved the quality of the manuscripts we publish and are critical to our journal’s success.

Thomaskutty Alumparambil, BS, CCP

Temple, TX, WA

Cory M Alwardt, PhD, CCP

Phoenix, AZ

Keith Amberman, BS, CCP

Olney, MD

Jennifer Baeza, MS, CCP

Tucson, AZ

Robert Baker, PhD, Dip Perf, CCP(AUST)

Adelaide, Australia

Jordan Brimhall, MS, CCP, FPP

Phoenix, AZ

Salman Pervaiz Butt, DBA, MBA, MSc, MPHYS

Abu Dhabi, UAE

Michael Colligan, DHA, RN, CCP

Plymouth, MI

Ignazio Condello, PhD 

Bari, Italy

Hamid Darban, PhD, CP

Glendale, AZ

Edward Darling, MS, CCP

Syracuse, NY

Bharat Datt, MSc, MBA, CCP, CPC, FPP, FAACP 

Orlando, FL

Filip M De Somer, PhD, ECCP

Gent, Belgium

Laura R Dell'Aiera, DHSc, CCP

Charleston, SC

Joseph J Deptula, MPS, CCP

Omaha, NE

Hongting Diao, PhD

People’s Republic of China

Molly Dreher, MA, CCP

Philadelphia, PA

Adam Fernandez, DHSc, MPS, CCP, LP

Gig Harbor, WA

Kenneth Fox, MD

Tucson, AZ

Larry Garrison, PhD, MBA, CCP 

Indianapolis, IN

Serdar Gunaydin, MD, PhD

Ankara, Turkey

Eugene A Hessel II, MD 

Lexington, KY

George Justison, CCP 

Denver, CO

Koichi Kashiwa, PhD 

Tokyo, Japan

Toshinobu Kazui, MD, PhD

Tucson, AZ

Robert Kramer, MD

Portland, ME

Supaporn Kulthinee, PhD

Nashville, TN

David Kwiatkowski, MD, MPH

Palo Alto, CA

Min-Ho Lee, PhD, CCP

Philadelphia, PA

Donald S Likosky, PhD

Ann Arbor, MI

Mark T Lucas, MPS, CCP 

Denver, CO

Kara Michele Lung, BS, CCP

Boston, MA

Annette Lisa Mazzone, PhD, Dip Perf, CCP(AUST)

Bedford Park, South Australia

Kevin H McCusker, PhD, CCP

Portsmouth, NH

Erick McNair, PhD, CCP

Atlanta, GA

Brian Mejak, CCP

Aurora, CO

HelenMari Merritt, DO 

Omaha, NE

Andrew D Meyer, MD, MS

San Antonio, TX

Linda B Mongero, BS, CCP Emeritus

New York, NY

Gerard J Myers, RT, CCP Emeritus

Halifax, Nova Scotia

James R Neal, CCP, FPP 

Rochester, MN

Kevin Niimi, MPS, CCP

Dallas, TX

Molly Elisabeth Oldeen, MS, CCP, FPP

Loiusville, KY

Jane Ottens, BSc, Dip Perf, CCP(AUST) 

Ashford, Australia

David A Palmer, EdD, CCP

Pittsburgh, PA 

Miguel Angel Parada-Nogueiras, PhD 

Leon, Spain

Giles Peek, MD

Gainesville, FL 

Thomas J Preston, MS, CCT 

Columbus, OH

Luc Puis, EBCP

Iowa City, IA

Thomas Rath, MPS, CP 

Glendale, AZ

Tami Rosenthal, MBA, CCP

Philadelphia, PA

Raya Safa, MD

Chicago, IL

Keith Samolyk, BS, RRT, CCP Emeritus, PBMS

Somers, CT

Jennifer Schaadt, MS, MBA

Lawrenceville, NJ

Kenneth G Shann, CCP

Boston, MA

David Andrew Sidebotham, MBChB, FANZCA

Auckland, NZ

Justin Roy Sleasman, MS, CCP 

Palo Alto, CA

Michael Smith, PhD, CCP

Hamden, CT

Alfred H Stammers, MSA, CCP Emeritus 

Sweet Valley, PA

Carrie Striker, DHEd, MPS, CCP, FPP

Milwaukee, WI

Nathaniel Sznycer-Taub, MD

Ann Arbour, MI

Tomohisa Takeichi, MA, CCP

Ono, Japan

Mukta Tiwari, PhD

Jaipur, India

Cody C Trowbridge, MPS, CCP

Danville, PA

Zasha Vazquez-Colon, MD 

Gainesville, FL

Kevin Watt, MD, PhD 

Salt Lake City, UT 

Patrick W Weerwind, PhD, CCP

Wijchen, The Netherlands

Julie Wegner, PhD

Tucson, AZ

